# Supplementary material for: Sintilimab with two cycles of chemotherapy for the treatment of advanced squamous non-small cell lung cancer: a phase 2 clinical trial
Source: Nat Commun. 2024 Feb 19;15:1512. doi: 10.1038/s41467-024-45769-z (PMC10876536; doi:10.1038/s41467-024-45769-z)
Supplement: Supplementary file 1 — Supplementary Information [file 41467_2024_45769_MOESM1_ESM.pdf]

Supplementary Information for

Sintilimab with two cycles of chemotherapy for the treatment of advanced squamous non-small cell lung cancer: a phase 2 clinical trial

Mina Zhang[1], Guowei Zhang[1], Yuanyuan Niu[1], Guifang Zhang[2], Yinghua Ji[3], Xiangtao Yan[1], Xiaojuan Zhang[1], Qichuan Wang[4], Xiaohui Jing[5], Junsheng Wang[6], Zhiyong Ma[1], Huijuan Wang[1,*]

[1]Department of Medical Oncology, The Affiliated Cancer Hospital of Zhengzhou University/Henan Cancer Hospital, 127 Dongming Rd, Zhengzhou 450003, China. [2]Department of Medical Oncology, Xin Xiang Central Hospital, 56 Jinsui Rd, Xinxiang 453000, China. [3]Department of Medical Oncology, The First Affiliated Hospital of Xin Xiang Medical University, 88 Jiankang Rd, Xinxiang 453199, China. [4]Department of Medical Oncology, The Second People's Hospital of Nan Yang, 66 Jianshe Rd, Nanyang 473000, China. [5]Department of Medical Oncology, The First People's Hospital of Ping Ding Shan, 117 Youyue Rd, Pingdingshan 467099, China. [6]Department of Medical Oncology, An Yang Cancer Hospital, 2 N Huanbin Rd, Anyang 455001, China.

***Corresponding author:**

Huijuan Wang

Department of Medical Oncology, The Affiliated Cancer Hospital of Zhengzhou University, Henan Cancer Hospital, 127 Dongming Rd, Zhengzhou 450003, China

Tel: +86-0371-65587013

Email: drwanghuijuan037@sina.com

CONTENTS LIST

Page 4: Supplementary Methods | Inclusion and exclusion criteria as well as any changes in enrolled patients before and after the protocol amendment

Page 9: Supplementary Table 1 | Reasons why patients did not undergo radical surgery, neoadjuvant treatment followed by surgery or PACIFIC regimens

Page 10: Supplementary Table 2 | Univariate Cox regression analysis of factors influencing PFS (efficacy analysis set, *n* = 44)

Page 12: Supplementary Table 3 | Univariate Cox regression analysis of factors influencing OS (efficacy analysis set, *n* = 44)

Page 14: Supplementary Table 4 | Comparison of ORR, PFS, OS (*n* = 44) and DOR (*n* = 31) between the subgroup populations with different PD-L1 expressions

Page 16: Supplementary Table 5 | Information of 6 participating sites

Page 17: Supplementary Fig. 1 | Kaplan-Meier curve of OS times for second line treatments.

Page 18: Supplementary Fig. 2 | Comparison of ORR, PFS, OS (*n* = 44) and DOR (*n* = 31) between the subgroup populations with different PD-L1 expressions.

Page 19: Supplementary Fig. 3 | Survival analysis for patients with or without *BRCA2*, *BRINP3*, *FBXW7*, *KIT* or *RB1* abnormalities (*n* = 26).

Page 20: Supplementary Fig. 4 | ROC curve for the prognostic efficacy of predictive biomarkers (*n* = 26).

Page 21: The translated simplified version of clinical trial protocol (No. CIBI308Y014)

Supplementary Methods | Inclusion and exclusion criteria as well as any changes of enrolled patients before and after the protocol amendment

Inclusion criteria:

Subjects who met the following enrollment criteria:

1. Male or female aged 18 to 75 years at the time of signing informed consent form.

2. Each subject voluntarily agreed to participate and signed an informed consent form.

3. Histologically or cytologically confirmed locally advanced or metastatic squamous NSCLC (unresectable or Stage IIIB-IV disease that did not meet the criteria for radical radiotherapy). Subjects with mixed squamous NSCLC with a > 50% squamous component were enrolled at the discretion of the investigator on a case-by-case basis.

4. Had received no prior systemic therapy for advanced or metastatic disease. Subjects who had received prior neoadjuvant/adjuvant therapy or chemoradiotherapy with curative intent, with confirmed disease recurrence at least 6 months after completion of the last treatment.

5. Were able to provide fresh or archived tumor tissue (formalin-fixed, paraffin-embedded [FFPE] tissue blocks or at least 10 unstained FFPE slides) and their pathology report. If a subject could provide < 10 unstained slides, enrollment was at the discretion of the investigator on a case-by-case basis.

6. The presence of at least one measurable lesion that met the RECIST 1.1 criteria of a non-lymph node lesion with a long diameter of ≥ 10 mm or a lymph node lesion with a short diameter ≥ 15 mm, based on CT cross-sectional images.

7. ECOG score of 0-1.

8. Expected survival of not less than 12 weeks.

9. Vital organ and bone marrow function met the following requirements:

Complete Blood Count (CBC): Absolute Neutrophil Count (ANC) ≥ 1.5 × 10^9/L, Platelet count (PLT) ≥ 100×10^9/L, Hemoglobin (HGB) ≥ 9 g/dL, (Note: These criteria must be met without receiving any blood transfusion within the 4 weeks prior to obtaining the blood sample). Liver function test (LFT): Serum total bilirubin (TBIL) ≤ 1.5 times the upper limit of normal (ULN); alanine aminotransferase (ALT) and/or aspartate aminotransferase (AST) ≤ 2.5 times the ULN; if the abnormal liver function is caused by hepatocellular carcinoma or tumor metastasis to the liver; AST and ALT ≤ 5 times the ULN; serum albumin (ALB) ≥ 2.8 g/dL; renal function test (RFT); serum creatinine (Cr) ≤ 1.5 times the ULN; or creatinine clearance ≥ 40 mL/min (using the standard Cockcroft-Gault formula).

10. Women of childbearing potential were required to use highly effective contraception during the study period, as well as for ≥ 120 days after the last sintilimab administration and for ≥ 180 days after the last chemotherapy treatment (see Appendix 4). Ideally, contraception measures should have been initiated at least 3 months before the first dose of the investigational treatment.

11. Non-sterilized males were required to use highly effective contraception for ≥ 180 days during the study period, and after the last sintilimab administration and the last chemotherapy treatment. It is recommended contraception be initiated at least 3 months prior to the first dose of the study treatment.

Exclusion criteria:

Subjects must not have any of the following exclusion criteria:

1. Known driver gene mutations (e.g., *EGFR*/*ROS1*/*ALK*, etc.) in lung squamous cell carcinoma.

2. Symptomatic central nervous system (CNS) metastases. However, for those with asymptomatic brain metastases or stable symptoms after treatment, they were eligible for the study if they met all of the following criteria: measurable lesions outside the CNS; no metastases in the midbrain, pons, cerebellum, meninges, medulla oblongata or spinal cord; and no requirement for hormone therapy with clinical stability for at least 2 weeks.

3. Had previously received anti-tumor treatment for other malignant tumors, including radiotherapy, chemotherapy, immunotherapy, and traditional Chinese medicine treatment (except for patients with curative treatment and no recurrence or metastasis for ≥ 5 years).

4. With uncontrollable pleural effusion, pericardial effusion or ascites that require repeated drainage (patients who do not need drainage or show no significant increase in fluid after drainage for 3 days may have been eligible for enrollment).

5. Had received immunosuppressive drugs within 4 weeks before the first dose of the investigational treatment, excluding topical or inhaled corticosteroids or physiologic doses of systemic corticosteroids (i.e., not exceeding 10 mg/day of prednisone or equivalent doses of other corticosteroids).

6. Known or suspected active autoimmune diseases (congenital or acquired), such as interstitial pneumonia, uveitis, enteritis, hepatitis, hypophysitis, vasculitis, nephritis, thyroiditis, etc. Patients with fully resolved childhood asthma or vitiligo, without any intervention in adulthood may be eligible for enrollment. Those with well-controlled type 1 diabetes patients on insulin therapy may also have been eligible.

7. Known allogeneic organ transplantation (except for corneal transplantation) or allogeneic hematopoietic stem cell transplantation.

8. Allergy to any component of the monoclonal antibody.

9. Current interstitial lung disease.

10. Patients with other uncontrolled severe diseases, including but not limited to:

1) Active or poorly controlled severe infections.

2) HIV-positive patients (positive for HIV antibodies).

3) Patients with acute or chronic active hepatitis B (positive for HBsAg and HBV DNA > 1×10^3/mL) or acute or chronic active hepatitis C (positive for HCV antibodies and HCV RNA > 15 IU/mL).

4) Active pulmonary tuberculosis, etc.

5) NYHA class III-IV congestive heart failure with poorly controlled and clinically significant arrhythmias.

6) Uncontrolled hypertension (systolic blood pressure ≥ 160 mmHg or diastolic blood pressure ≥ 100 mmHg).

7) Any arterial thrombosis, embolism, or ischemic event (e.g., myocardial infarction, unstable angina, cerebrovascular accident or transient ischemic attack) occurring within 6 months prior to study enrollment.

8) Diseases requiring the use of warfarin (coumarin) anticoagulation therapy.

9) Uncontrolled hypercalcemia (calcium ion greater than 1.5 mmol/L or calcium greater than 12 mg/dL or corrected serum calcium greater than ULN) or symptomatic hypercalcemia requiring continued bisphosphonate treatment.

10) Concurrently diagnosed with other malignancies (excluding those that have been curatively treated, such as in situ cervical cancer, non-melanoma skin cancer, etc.).

11. Other acute or chronic diseases, psychiatric disorders, or abnormal laboratory values that may increase the risks associated with study participation or the administration of study drugs, and interfere with the interpretation of study results, as determined by the investigator.

12. Pregnant or lactating females.

13. Received prophylactic vaccines against infections (such as influenza virus vaccine, human papillomavirus vaccine) within 4 weeks before the initiation of the investigational treatment. During the treatment period, the use of all other vaccines, except for non-live vaccines, was prohibited.

14. Had underwent major surgery within 4 weeks (28 days) prior to the initiation of study drug administration, except for surgery performed for diagnostic purposes.

15. Subjects for whom the investigator deems the paclitaxel (or nab-paclitaxel)/cisplatin (or carboplatin) chemotherapy regimen inappropriate for them.

Any changes of enrolled patients before and after the protocol amendment

Before the revision of the protocol in April 2020, one patient (01001) was enrolled and randomized to the control group receiving chemotherapy alone, with the informed consent signed on September 3, 2019. After the protocol revision, this patient was not included in the study as for dissatisfaction of the criteria of the revised protocol. Following the revision to version 2.1 of the protocol, the first patient to sign informed consent was assigned to the identifier 01002 on May 4, 2020.

Supplementary Table 1 | Reasons why patients did not undergo radical surgery, neoadjuvant treatment followed by surgery or PACIFIC regimens

Number	Clinical stage	Reason
01002	IIIC	Extensive cancer-associated lymphangitis
01009	IIIB	Huge tumor mass, and the boundary between the mass and the oesophagus is unclear
01011	IIIC	High tumor burden, extensive lymph node involvement, low lung function, high risk for radiotherapy
01013	IIIC	Multiple nodules in different lung lobes on the same side
01022	IIIC	Bilateral enlargement of hilar lymph nodes, unsuitable for radiotherapy
01025	IIIC	The patient declined radiotherapy
01029	IIIB	Bilateral enlargement of hilar lymph nodes, unsuitable for radiotherapy
01030	IIIB	Satellite lesions scattering in the same lung lobe, unsuitable for radiotherapy
01034	IIIC	An extensive tumor (10.5 cm) involving left main bronchus and causing complete lung collapse
03003	IIIB	A large cystic lesion containing an air-fluid level
06001	IIIB	Recurrent left lung cancer invading the left and right main bronchus, high risk for radiotherapy

Supplementary Table 2 | Univariate Cox regression analysis of factors influencing PFS (efficacy analysis set, *n* = 44)

	Case/*n*	Univariate HR (95% CI)	*Wald Chi-square*	*P*-value
Age				
< 65	12/19	Ref		
≥ 65	17/25	1.42 (0.68, 2.99)	0.86	0.354
Smoking status				
Never	4/8	Ref		
Current/ Former	25/36	1.71 (0.60, 4.93)	0.99	0.319
ECOG PS				
0	10/13	Ref		
1	19/31	0.65 (0.30, 1.40)	1.22	0.269
Clinical stage				
IIIB/IIIC	6/10	Ref		
IV	23/34	1.44 (0.58, 3.55)	0.62	0.432
Pathologic subtypes				
Mixed squamous NSCLC	2/3	Ref		
Squamous	27/41	1.43 (0.34, 6.10)	0.24	0.625
PD-L1				
TPS < 1%	14/19	Ref		

TPS 1-49%	6/11	0.52 (0.20, 1.37)	1.73	0.188
TPS ≥ 50%	2/3	1.10 (0.25, 4.93)	0.02	0.896
ND	7/11	0.69 (0.28, 1.73)	0.62	0.431

Univariate Cox regression analysis was performed two sided. All degrees of freedom are 1.

CI confidence interval, *ECOG PS* Eastern Cooperative Oncology Group Performance Status, *HR* hazard ratio, *ND* not detectable, *NSCLC* non-small cell lung cancer, *PD-L1* programmed cell death ligand-1, *TPS* tumor cell proportion score.

Source data are provided in the Source Data file.

Supplementary Table 3 | Univariate Cox regression analysis of factors influencing OS (efficacy analysis set, *n* = 44)

	Case/*n*	Univariate HR (95% CI)	*Wald Chi-square*	*P*-value
Age				
< 65	6/19	Ref		
≥ 65	14/25	2.26 (0.86, 5.92)	2.74	0.098
Smoking status				
Never	4/8	Ref		
Current/ Former	16/36	0.79 (0.26, 2.38)	0.17	0.676
ECOG PS				
0	6/13	Ref		
1	14/31	0.87 (0.33, 2.31)	0.08	0.782
Clinical stage				
IIIB/IIIC	4/10	Ref		
IV	16/34	1.37 (0.46, 4.10)	0.31	0.578
Pathologic subtypes				
Mixed squamous	1/3	Ref		
NSCLC				
Squamous	19/41	1.99 (0.26, 14.97)	0.44	0.505
PD-L1				
TPS < 1%	10/19	Ref		
TPS 1-49%	4/11	0.63 (0.20, 2.01)	0.61	0.433

TPS ≥ 50%	1/3	1.03 (0.13, 8.20)	< 0.01	0.981
ND	5/11	0.95 (0.32, 2.77)	0.01	0.920

Univariate Cox regression analysis was performed two sided. All degrees of freedom are 1.

CI confidence interval, *ECOG PS* Eastern Cooperative Oncology Group Performance Status, *HR* hazard ratio, *ND* not detectable, *NSCLC* non-small cell lung cancer, *PD-L1* programmed cell death ligand-1, *TPS* tumor cell proportion score.

Source data are provided in the Source Data file.

Supplementary Table 4 | Comparison of ORR, PFS, OS (*n* = 44) and DOR (*n* = 31) between the subgroup populations with different PD-L1 expressions

	TPS < 1% *n* = 19	TPS = 1–49% *n* = 11	TPS ≥ 50% *n* = 3	*Chi-square*	*P*-value (< 1% vs. 1–49% vs. ≥ 50%)	PD-L1 ND *n* = 11	*Chi-square*	*P*-value (for 4 groups)
ORR (%)	63.2	81.8	66.7	/	0.634	72.7	/	0.770
PFS (m), median (95% CI)	10.1 (4.2–19.9)	11.5 (3.2–NE)	5.2 (4.2–NE)	1.87	0.393	11.9 (6.5–NE)	2.15	0.542
OS (m), median (95% CI)	21.9 (16.4–NE)	NE (8.8–NE)	NE (8.1–NE)	0.62	0.733	27.2 (8.5–NE)	0.66	0.882

DOR (m),	*n* =12	*n* = 9	*n* = 2			*n* = 8		
median	16.4 (2.8–19.7)	NE (4.0–NE)		15.92	< 0.001	12.1 (4.8–NE)	22.24	< 0.001
(95% CI)			2.5 (2.2–2.8)					

The Fisher's exact test was performed to compare ORR, and the log-rank test was employed to compare PFS, OS and DOR. Both statistical tests are two sided. The degrees of freedom of three groups comparison are 2, of four groups comparison are 3.

CI confidence interval, *DOR* duration of response, *HR* hazard ratio, *m* month, *ND* not detectable, *NE* not evaluable, *ORR* objective response rate, *OS* overall survival, *PFS* progression free survival, *TPS* tumor cell proportion score.

Source data are provided in the Source Data file.

Supplementary Table 5 | Information of 6 participating sites

Site No.	Institution	City	No. of enrolled patients
01	The Affiliated Cancer Hospital of Zhengzhou University/Henan Cancer Hospital	Zhengzhou	37
02	The First Affiliated Hospital of Xin Xiang Medical University	Xinxiang	3
03	Xin Xiang Central Hospital	Xinxiang	3
04	The First People’s Hospital of Ping Ding Shan	Pingdingshan	2
05	An Yang Cancer Hospital	Anyang	1
06	The Second People’s Hospital of Nan Yang	Nanyang	2

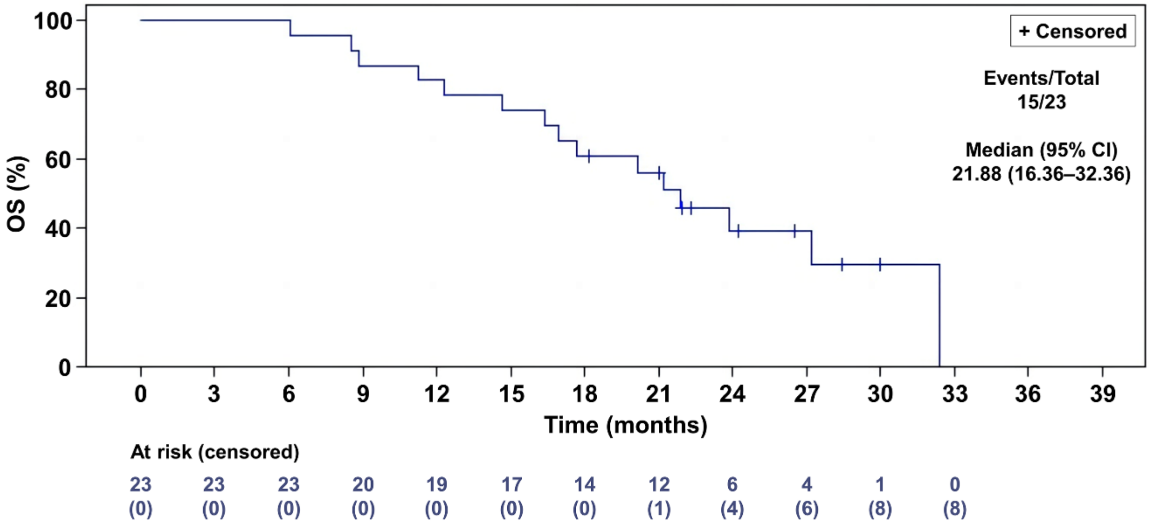

Supplementary Fig. 1 | Kaplan-Meier curve of OS times for second line treatments.

CI confidence interval, *OS* overall survival.

Source data are provided in the Source Data file.

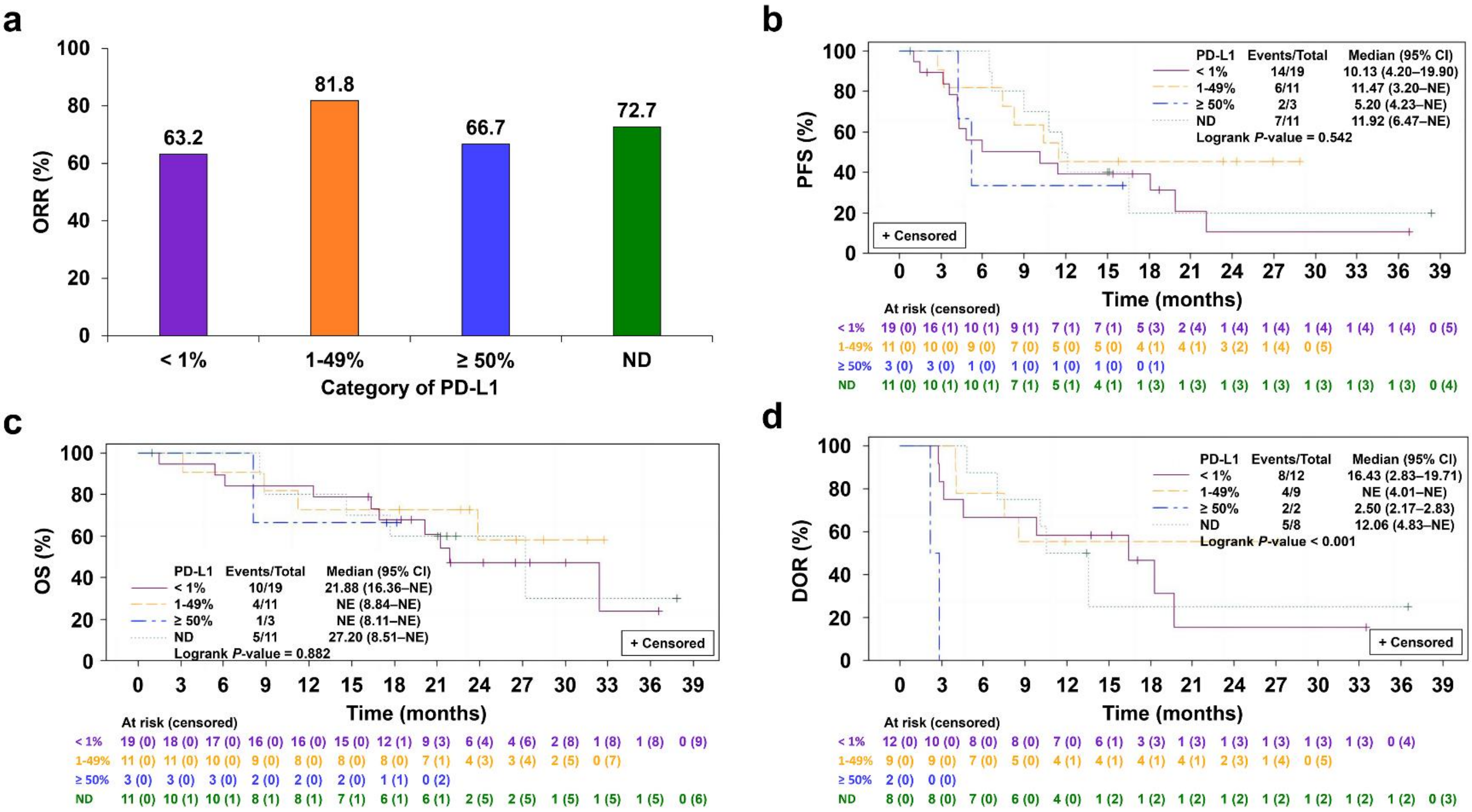

Supplementary Fig. 2 | Comparison of ORR, PFS, OS (*n* = 44) and DOR (*n* = 31) between the subgroup populations with different PD-L1 expressions.

In panels **a-c**, TPS < 1%, *n* = 19, TPS = 1–49%, *n* = 11, TPS ≥ 50%, *n* = 3, ND, *n* = 11. In panel **d**, TPS < 1%, *n* = 12, TPS = 1–49%, *n* = 9, TPS ≥ 50%, *n* = 2, ND, *n* = 8.

b-d *P*-values were calculated using the two-sided log-rank test at a significance level of 0.05, and no adjustments were made for multiplicity. Source data are provided as a Source Data file. **b** Chi-square = 2.15. **c** Chi-square = 0.66. **d** Chi-square =22.24. All degrees of freedom are 3.

CI confidence interval, *DOR* duration of response, *HR* hazard ratio, *m* month, *ND* not detectable, *NE* not evaluable, *ORR* objective response rate, *OS* overall survival, *PFS* progression free survival.

Source data are provided in the Source Data file.

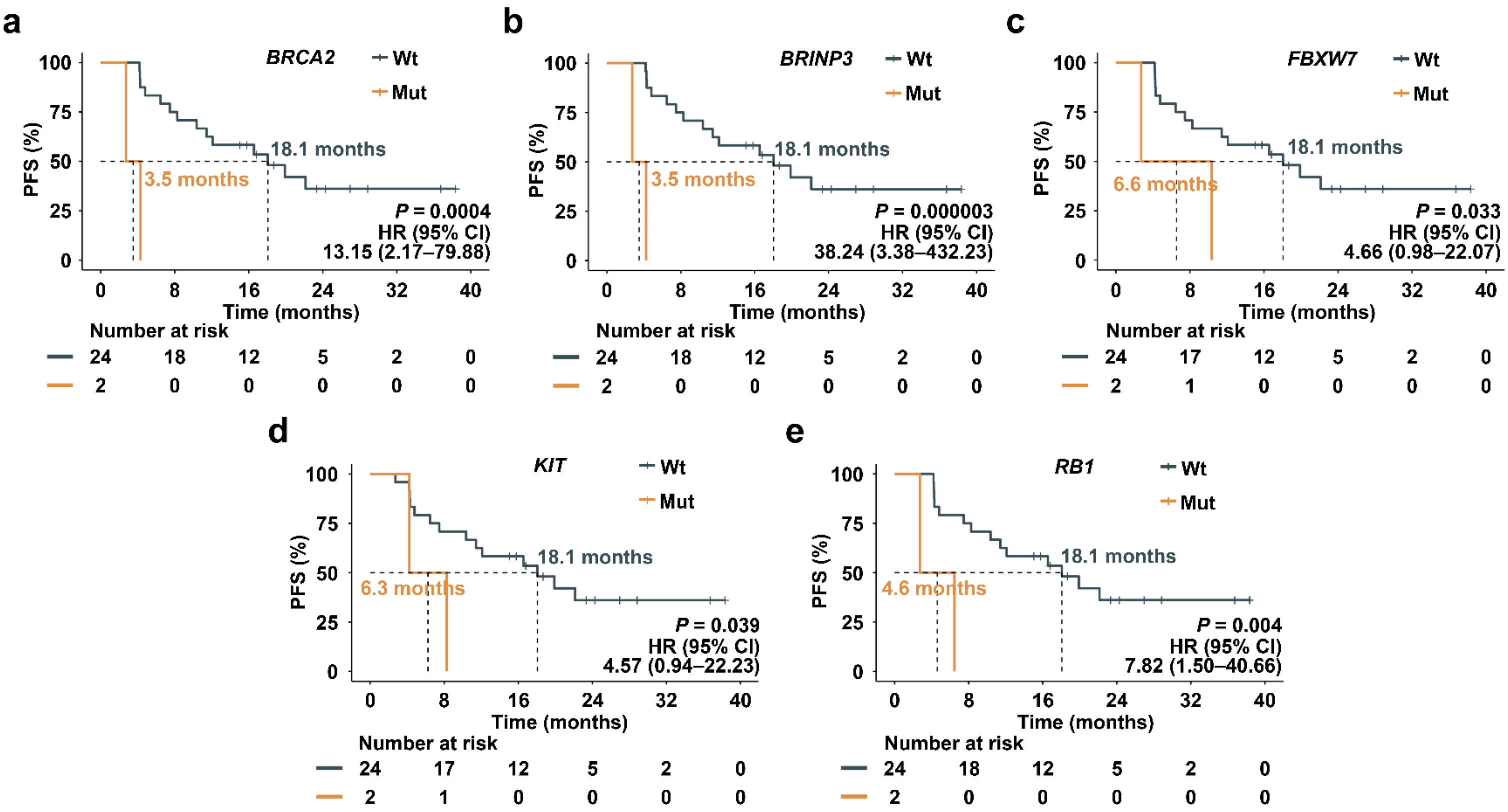

Supplementary Fig. 3 | Survival analysis for patients with or without *BRCA2, BRINP3, FBXW7, KIT* or *RB1* abnormalities (*n* = 26).

a-e *P*-values were calculated using the one-sided log-rank test at a significance level of 0.05, and no adjustments were made for multiplicity.

BRCA2 breast cancer gene 2, *BRINP3* BMP/Retinoic Acid Inducible Neural Specific 3, *CI* confidence interval, *ctDNA* circulating tumor DNA, *FBXW7* F-box/WD repeat-containing protein 7, *HR* hazard ratio, *KIT* tyrosine-protein kinase KIT, *Mut* mutation, *PFS* progression-free survival, *RB1* retinoblastoma 1, *Wt* wild type.

Source data are provided in the Source Data file.

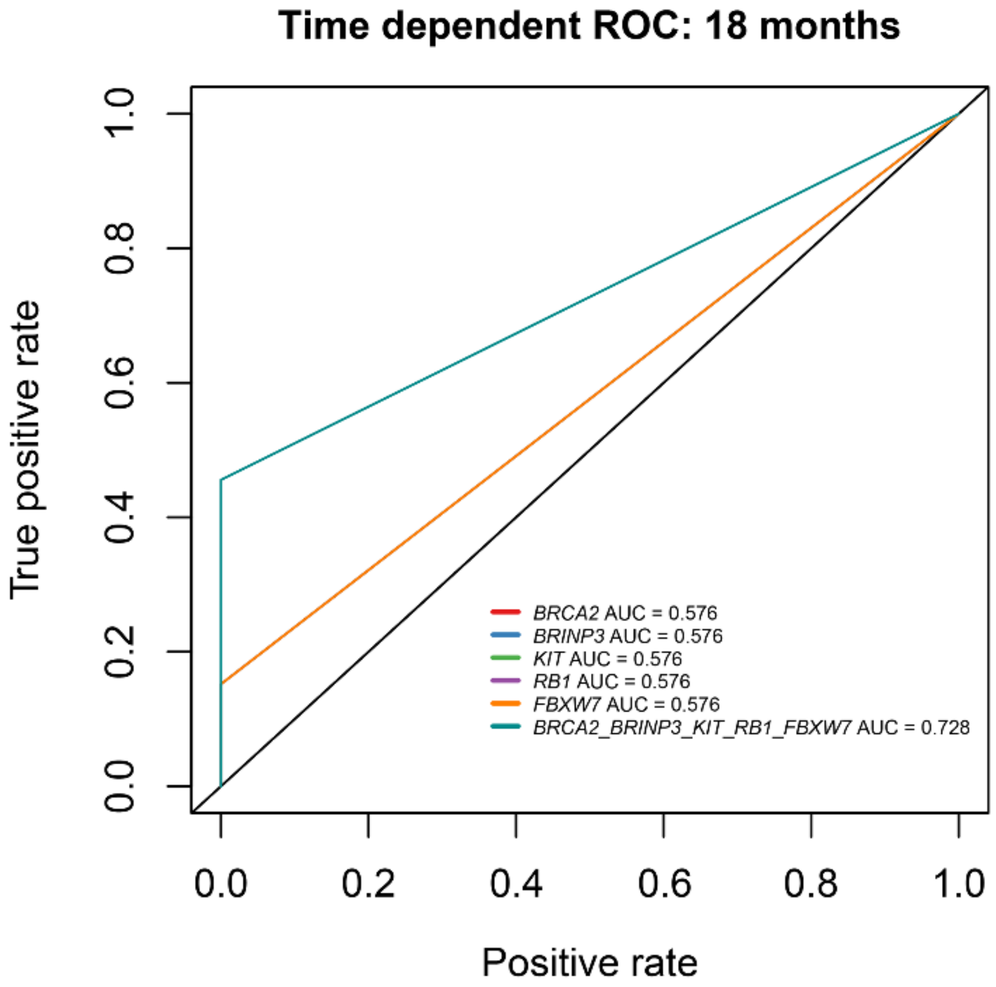

Supplementary Fig. 4 | ROC curve for the prognostic efficacy of predictive biomarkers (*n* = 26).

BRCA2 breast cancer gene 2, *BRINP3* BMP/Retinoic Acid Inducible Neural Specific 3, *FBXW7* F-box/WD repeat-containing protein 7, *KIT* tyrosine-protein kinase KIT, *RB1* retinoblastoma 1.

The translated simplified version of clinical trial protocol (No. CIBI308Y014)

The protocol title

Anti PD-1 inhibitor Sintilimab combined with short course of nab-paclitaxel/platinum as first line therapy for locally advanced or metastatic squamous non-small cell lung cancer: a phase 2 clinical trial

Protocol version and modification

V2.1, April 26, 2020

This was a prospective, multi-center, open-label, single-arm, phase 2 clinical trial, which was designed and registered as version 1.0 on December 26, 2018 with the protocol of a randomized controlled phase 2 clinical study of a short-course chemotherapy of paclitaxel/platinum with sintilimab versus paclitaxel/platinum as first-line treatment for locally advanced or metastatic sq-NSCLC in Chinese patients. Since PD-1 inhibitors in combination with chemotherapy became the standard treatment for advanced sq-NSCLC in 2019, a chemotherapy arm does not meet the patient's needs for treatment anymore. Therefore we modified the protocol (V2.1, April 26, 2020) to single-arm clinical trial of sintilimab combined with two cycles (short-course) nab-paclitaxel/platinum as first-line therapy for locally advanced or metastatic sq-NSCLC in a phase 2, single-arm clinical study.

Registration number

ChiCTR1900021726

Trial design

This is a multicenter phase 2 study to evaluate the efficacy of the anti-PD-1 monoclonal

antibody Sintilimab combined with an albumin-paclitaxel plus platinum-based short-course chemotherapy for the treatment of locally advanced or metastatic lung squamous cell carcinoma in China.

Subjects received the following treatments on day 1 of every 21 days (3 weeks): Sintillimab 200 mg, albumin paclitaxel 260 mg/m^2, cisplatin 75mg/m^2 (or carboplatin AUC = 5), IV (intravenous). Albumin-paclitaxel + cisplatin (or carboplatin) treatment will last for 2 cycles. After 2 cycles, patients who did not progress as assessed were continued to be treated with sintilimab maintenance therapy every 3 weeks until disease progression assessed by RECIST 1.1, or there was no benefit from treatment, or intolerable toxicity, or withdrawal of consent.

The study process included a screening period (within 28 days before the start of treatment), a treatment period (until the subject's disease progresses, the investigator's judgment was unable to see a benefit from treatment, intolerable toxicity occured, or the subject withdrawed informed consent, etc.), and a safety follow-up period (within 30 days after the last study treatment) and survival follow-up period.

Tumor response was evaluated by investigators based on Solid Tumor Response Evaluation Criteria (RECIST) version 1.1 every 6 weeks in the first year and every 12 weeks after the first year until progression. If a subject discontinued the study treatment for reasons other than disease progression or death, tumor evaluation should continue as planned until a new anticancer treatment has been initiated, disease progression, death, loss of follow-up, or consent has been withdrawn.

Endpoints

The primary endpoint was progression-free survival (PFS, defined as the time from the

date of signing informed consent to the date of disease progression or death).

The secondary endpoints included the objective response rate (ORR); disease control rate (DCR); duration of response (DOR); OS (time from receipt of signed informed content to death or the last follow-up); and safety. Tumour responses were assessed by the investigator according to the Response Evaluation Criteria in Solid Tumors (RECIST) version 1.1 (1) every 6 weeks in the first year and every 12 weeks there after until disease progression. Safety was measured by the prevalence of treatment-related adverse events, which were graded based on NCI Common Terminology Criteria for Adverse Events (CTCAE) version 5.0. Changes in vital signs, physical examination results, and laboratory tests (such as hematology, clinical biochemistry, urine analysis) before, during and after treatment were carefully recorded and assessed.

Exploratory study endpoint: To evaluate the relationship between biomarkers of tumor tissues and efficacy, including but not limited to PD-L1 expression, tumor infiltrating lymphocytes (TILs), and tumor mutational burden (TMB). To evaluate the relationship between biomarkers in peripheral blood and efficacy, including but not limited to the relationship between dynamic changes in ctDNA mutation load and efficacy. To evaluate the possible effects of dynamic changes in peripheral blood biomarkers before and after sintilimab treatment on the tumor microenvironment, including but not limited to T cell count, activation, proliferation, γ-interferon and γ-interferon-associated chemokines.

Statistical consideration

Sample size

In this study PFS was used as a primary endpoint and the objective was to investigate whether a short course of chemotherapy combined with immunotherapy could prolong PFS

in sq-NSCLC patients. According to published data, the median PFS for 4-6 cycles of a previously applied control chemotherapy with a paclitaxel/cisplatin (or carboplatin) regimen was 4.4 months (2-3) and it has been hypothesized that the addition of maintenance therapy with sintilimab after 2 cycles of sintilimab plus paclitaxel (nab-paclitaxel)/cisplatin (or carboplatin) would yield a 45% PFS improvement, resulting in a duration of 6.4 months as compared to the published median PFS time. To assess this hypothesis, the HR of $\lambda 1/\lambda 0$ was approximately 0.69 by using a log rank test with a single bias type I error of 0.10, followed by a power of 80%. With an expected enrolment time of at least 12 months, and a follow-up time of 12 months, a minimum of 41 patients were needed to be enrolled to observe 34 events. Considering a 20% dropout rate, a total of 50 patients were enrolled in this study.

Analysis set

All analyses were based on the ITT (intention-to-treat) principle. Patients in the **efficacy analysis set** received at least one dose of sintilimab and/or chemotherapy and had at least one tumour response evaluation. **The safety analysis set** refers to patients who received at least one dose of sintilimab and/or chemotherapy.

Analysis plan

All statistical analyses were conducted using SAS 9.4 (SAS Institute, Inc., Cary, North Carolina). The median PFS, OS and DOR were estimated by the Kaplan-Meier (K-M) method, and their 95% confidence intervals (CIs) evaluated using the Greenwood formula. A Cox regression model was used to estimate the treatment HR and the impact of the analyzed variables on PFS and OS.

Inclusion criteria:

1. Male or female aged 18 to 75 years at the time of signing informed consent form.

2. Each subject voluntarily agreed to participate and signed an informed consent form.

3. Histologically or cytologically confirmed locally advanced or metastatic squamous NSCLC (unresectable or Stage IIIB-IV disease that did not meet the criteria for radical radiotherapy). Subjects with mixed squamous NSCLC with a > 50% squamous component were enrolled at the discretion of the investigator on a case-by-case basis.

4. Had received no prior systemic therapy for advanced or metastatic disease. Subjects who had received prior neoadjuvant/adjuvant therapy or chemoradiotherapy with curative intent, with confirmed disease recurrence at least 6 months after completion of the last treatment.

5. Were able to provide fresh or archived tumor tissue (formalin-fixed, paraffin-embedded [FFPE] tissue blocks or at least 10 unstained FFPE slides) and their pathology report. If a subject could provide < 10 unstained slides, enrollment was at the discretion of the investigator on a case-by-case basis.

6. The presence of at least one measurable lesion that met the RECIST 1.1 criteria of a non-lymph node lesion with a long diameter of ≥ 10 mm or a lymph node lesion with a short diameter ≥ 15 mm, based on CT cross-sectional images.

7. ECOG score of 0-1.

8. Expected survival of not less than 12 weeks.

9. Vital organ and bone marrow function met the following requirements:

Complete Blood Count (CBC): Absolute Neutrophil Count (ANC) ≥ 1.5 × 10^9/L, Platelet count (PLT) ≥ 100×10^9/L, Hemoglobin (HGB) ≥ 9 g/dL, (Note: These criteria must be met without receiving any blood transfusion within the 4 weeks prior to obtaining the blood

sample). Liver function test (LFT): Serum total bilirubin (TBIL) ≤ 1.5 times the upper limit of normal (ULN); alanine aminotransferase (ALT) and/or aspartate aminotransferase (AST) ≤ 2.5 times the ULN; if the abnormal liver function is caused by hepatocellular carcinoma or tumor metastasis to the liver; AST and ALT ≤ 5 times the ULN; serum albumin (ALB) ≥ 2.8 g/dL; renal function test (RFT); serum creatinine (Cr) ≤ 1.5 times the ULN; or creatinine clearance ≥ 40 mL/min (using the standard Cockcroft-Gault formula).

10. Women of childbearing potential were required to use highly effective contraception during the study period, as well as for ≥ 120 days after the last sintilimab administration and for ≥ 180 days after the last chemotherapy treatment (see Appendix 4). Ideally, contraception measures should have been initiated at least 3 months before the first dose of the investigational treatment.

11. Non-sterilized males were required to use highly effective contraception for ≥ 180 days during the study period, and after the last sintilimab administration and the last chemotherapy treatment. It is recommended that contraception be initiated at least 3 months prior to the first dose of the study treatment.

Exclusion criteria:

Subjects must not have any of the following exclusion criteria:

1. Known driver gene mutations (e.g., *EGFR/ROS1/ALK*, etc.) in lung squamous cell carcinoma.

2. Symptomatic central nervous system (CNS) metastases. However, for those with asymptomatic brain metastases or stable symptoms after treatment, they were eligible for the study if they met all of the following criteria: measurable lesions outside the CNS; no metastases in the midbrain, pons, cerebellum, meninges, medulla oblongata or spinal cord;

and no requirement for hormone therapy with clinical stability for at least 2 weeks.

3. Had previously received anti-tumor treatment for other malignant tumors, including radiotherapy, chemotherapy, immunotherapy, and traditional Chinese medicine treatment (except for patients with curative treatment and no recurrence or metastasis for ≥ 5 years).

4. With uncontrollable pleural effusion, pericardial effusion or ascites that require repeated drainage (patients who do not need drainage or show no significant increase in fluid after drainage for 3 days may have been eligible for enrollment).

5. Had received immunosuppressive drugs within 4 weeks before the first dose of the investigational treatment, excluding topical or inhaled corticosteroids or physiologic doses of systemic corticosteroids (i.e., not exceeding 10 mg/day of prednisone or equivalent doses of other corticosteroids).

6. Known or suspected active autoimmune diseases (congenital or acquired), such as interstitial pneumonia, uveitis, enteritis, hepatitis, hypophysitis, vasculitis, nephritis, thyroiditis, etc. Patients with fully resolved childhood asthma or vitiligo, without any intervention in adulthood may be eligible for enrollment. Those with well-controlled type 1 diabetes patients on insulin therapy may also have been eligible.

7. Known allogeneic organ transplantation (except for corneal transplantation) or allogeneic hematopoietic stem cell transplantation.

8. Allergy to any component of the monoclonal antibody.

9. Current interstitial lung disease.

10. Patients with other uncontrolled severe diseases, including but not limited to:

1) Active or poorly controlled severe infections.

2) HIV-positive patients (positive for HIV antibodies).

3) Patients with acute or chronic active hepatitis B (positive for HBsAg and HBV DNA > 1×10^3/mL) or acute or chronic active hepatitis C (positive for HCV antibodies and HCV RNA > 15 IU/mL).

4) Active pulmonary tuberculosis, etc.

5) NYHA class III-IV congestive heart failure with poorly controlled and clinically significant arrhythmias.

6) Uncontrolled hypertension (systolic blood pressure ≥ 160 mmHg or diastolic blood pressure ≥ 100 mmHg).

7) Any arterial thrombosis, embolism, or ischemic event (e.g., myocardial infarction, unstable angina, cerebrovascular accident or transient ischemic attack) occurring within 6 months prior to study enrollment.

8) Diseases requiring the use of warfarin (coumarin) anticoagulation therapy.

9) Uncontrolled hypercalcemia (calcium ion greater than 1.5 mmol/L or calcium greater than 12 mg/dL or corrected serum calcium greater than ULN) or symptomatic hypercalcemia requiring continued bisphosphonate treatment.

10) Concurrently diagnosed with other malignancies (excluding those that have been curatively treated, such as in situ cervical cancer, non-melanoma skin cancer, etc.).

11. Other acute or chronic diseases, psychiatric disorders, or abnormal laboratory values that may increase the risks associated with study participation or the administration of study drugs, and interfere with the interpretation of study results, as determined by the investigator.

12. Pregnant or lactating females.

13. Received prophylactic vaccines against infections (such as influenza virus vaccine,

human papillomavirus vaccine) within 4 weeks before the initiation of the investigational treatment. During the treatment period, the use of all other vaccines, except for non-live vaccines, was prohibited.

14. Had underwent major surgery within 4 weeks (28 days) prior to the initiation of study drug administration, except for surgery performed for diagnostic purposes.

15. Subjects for whom the investigator deems the paclitaxel (or nab-paclitaxel)/cisplatin (or carboplatin) chemotherapy regimen inappropriate.

Important date

Start date: September 4, 2019

Date of LPI (last patient in): April 25, 2022

Data cut off: August 31, 2023

Median follow-up: 24.2 months (range: 1.0-37.8)

As of August 31, 2023, there were still 2 patients in treatment and follow-up.
