# Supplementary material for: Sintilimab with two cycles of chemotherapy for the treatment of advanced squamous non-small cell lung cancer: a phase 2 clinical trial
Source: Nat Commun. 2024 Feb 19;15:1512. doi: 10.1038/s41467-024-45769-z (PMC10876536; doi:10.1038/s41467-024-45769-z)
Supplement: Supplementary file 3 — Reporting Summary [file 41467_2024_45769_MOESM3_ESM.pdf]

nature portfolio

Corresponding author(s): Huijuan Wang

Last updated by author(s): Feb 4, 2024

Reporting Summary

Nature Portfolio wishes to improve the reproducibility of the work that we publish. This form provides structure for consistency and transparency in reporting. For further information on Nature Portfolio policies, see our Editorial Policies and the Editorial Policy Checklist.

Please do not complete any field with "not applicable" or n/a. Refer to the help text for what text to use if an item is not relevant to your study.
For final submission: please carefully check your responses for accuracy; you will not be able to make changes later.

Statistics

For all statistical analyses, confirm that the following items are present in the figure legend, table legend, main text, or Methods section.

n/a	Confirmed	
☐	☒	The exact sample size (*n*) for each experimental group/condition, given as a discrete number and unit of measurement
☒	☐	A statement on whether measurements were taken from distinct samples or whether the same sample was measured repeatedly
☐	☒	The statistical test(s) used AND whether they are one- or two-sided<br>*Only common tests should be described solely by name; describe more complex techniques in the Methods section.*
☐	☒	A description of all covariates tested
☒	☐	A description of any assumptions or corrections, such as tests of normality and adjustment for multiple comparisons
☐	☒	A full description of the statistical parameters including central tendency (e.g. means) or other basic estimates (e.g. regression coefficient) AND variation (e.g. standard deviation) or associated estimates of uncertainty (e.g. confidence intervals)
☐	☒	For null hypothesis testing, the test statistic (e.g. *F*, *t*, *r*) with confidence intervals, effect sizes, degrees of freedom and *P* value noted<br>*Give P values as exact values whenever suitable.*
☒	☐	For Bayesian analysis, information on the choice of priors and Markov chain Monte Carlo settings
☒	☐	For hierarchical and complex designs, identification of the appropriate level for tests and full reporting of outcomes
☐	☒	Estimates of effect sizes (e.g. Cohen's *d*, Pearson's *r*), indicating how they were calculated

Our web collection on statistics for biologists contains articles on many of the points above.

Software and code

Policy information about availability of computer code

Data collection	The data collection was conducted using 91Trial software version 1.0 (EDC system, Shanghai Ashermed Healthcare Communications Co., Ltd, Shanghai, China).
Data analysis	All statistical analyses were performed using R software (R version 4.1.2) and SAS 9.4 (SAS Institute Inc., Cary, NC). The Sankey figure was plotted using an online visualization tool (https://sankeymatic.com/build/). The genomic alteration figure was plotted using ComplexHeatmap package (Ref 48). The ROC curve was plotted using pROC package (Ref 49).

For manuscripts utilizing custom algorithms or software that are central to the research but not yet described in published literature, software must be made available to editors and reviewers. We strongly encourage code deposition in a community repository (e.g. GitHub). See the Nature Portfolio guidelines for submitting code & software for further information.

Data

Policy information about availability of data

All manuscripts must include a data availability statement. This statement should provide the following information, where applicable:

- Accession codes, unique identifiers, or web links for publicly available datasets
- A description of any restrictions on data availability
- For clinical datasets or third party data, please ensure that the statement adheres to our policy

The minimum datasets necessary to interpret this research have been provided within the Article, Supplementary Information and Source Data File, where

applicable. ctDNA sequencing data are available at the GSA-Human dataset HRA006484 (https://ngdc.cncb.ac.cn/gsa-human/browse/HRA006484) under restricted access. Access can be granted through the Data Access Committees (DAC) of the GSA-Human database (2024BAT00029), with an estimated response time of about five working days for access requests. Upon approval, the data will be accessible for a duration of six months. Applicants may also directly contact the corresponding author, Huijuan Wang. The original study protocol was written in Chinese, but a translated simplified version is available in the Supplementary Information file. Source data, including individual de-identified participant data, are provided in this paper.

Research involving human participants, their data, or biological material

Policy information about studies with human participants or human data. See also policy information about sex, gender (identity/presentation), and sexual orientation and race, ethnicity and racism.

Reporting on sex and gender	Sex was self-reported as part of patient demographics. The study included mostly males, which reflects the general sex disparity of sq-NSCLC in non-smoking Chinese since previous studies found that squamous NSCLC (sq-NSCLC) is the major histopathological cancer type in males, with a 5.7 times higher incidence in males than in females in Chinese non-smokers. In addition, previous research also indicated a stronger correlation of smoking with squamous cell cancer than with adenocarcinoma, while smoking is extremely unpopular for Chinese women.
Reporting on race, ethnicity, or other socially relevant groupings	Since the study has been conducted in China the majority of patients belonged to the Han ethnicity.
Population characteristics	Between May 2020 and June 2022, 48 patients signed informed consent, and 47 received at least one treatment. In total 44 patients had evaluable imaging data and 3 were excluded from the efficacy analysis (1 due to protocol deviation, 1 who withdrew consent and 1 who died of fungal pneumonia after 1 cycle of treatment) (Fig. 1). At the cut-off date (August 31, 2023), 4 (9.1%) patients were still receiving treatment, while 40 discontinued treatment, including 27 who had disease progression (PD), 4 with intolerable adverse events (AEs), 1 accidental death (not due to disease), 4 voluntary withdrawals, 1 lost to follow-up, and 3 who completed 2 years of treatment.The enrolled patients' median age was 65 years (range: 52–76). Most patients were male (45/46, 97.8%), smokers (37/46, 80.4%), had an Eastern Cooperative Oncology Group Performance Status (ECOG PS) score of 1 (32/46, 69.6%) and stage IV disease (35/46, 76.1%). Thirty-three patients had evaluable programmed cell death ligand-1 (PD-L1) expression results and 30.4% patients had a score ≥ 1%.
Recruitment	This trial was conducted at 6 Chinese centers between May 08, 2020 and August 31, 2023. The recruitment included advanced sq-NSCLC patients admitted to the participant centers, who met the inclusion criteria, ensuring the absence of selection bias. Trained clinicians at the participant centers conducted the initial screening, while the principal investigators were responsible for evaluating pretreatment assessment and determining eligibility. Written informed consent was obtained from all participants before screening.
Ethics oversight	The protocols were conducted in accordance with the Declaration of Helsinki and Good Clinical Practice guidelines and approved by each participating institutions ethics review board of 6 study sites (Supplementary File 2). All patients signed written informed consent before participating in the trial.

Note that full information on the approval of the study protocol must also be provided in the manuscript.

Field-specific reporting

Please select the one below that is the best fit for your research. If you are not sure, read the appropriate sections before making your selection.

☒ Life sciences ☐ Behavioural & social sciences ☐ Ecological, evolutionary & environmental sciences

For a reference copy of the document with all sections, see nature.com/documents/nr-reporting-summary-flat.pdf

Life sciences study design

All studies must disclose on these points even when the disclosure is negative.

Sample size	According to published data, the median PFS for 4–6 cycles of the historic control chemotherapy with a paclitaxel/cisplatin (or carboplatin) regimen was 4.4 months (ref 22, 48) and it has been hypothesized that the addition of maintenance therapy with sintilimab after 2 cycles of sintilimab plus paclitaxel (nab-paclitaxel)/cisplatin (or carboplatin) would yield a 45% PFS improvement, resulting in a duration of 6.4 months as compared to the published median PFS time. To assess this hypothesis, the HR of $\lambda 1/\lambda 0$ was approximately 0.69 by using a log rank test with a single bias type I error of 0.10, followed by a power of 80%. With an expected enrolment time of at least 12 months, and a follow-up time of 12 months, a minimum of 41 patients were needed to be enrolled to observe 34 events. Considering a 20% dropout rate, a total of 50 patients were enrolled in this study.
Data exclusions	Among the 48 enrolled patients, two were excluded from the data analysis—one was recruited before the protocol modification, and the other did not meet the eligibility criteria.
Replication	The findings in the present phase 2 trial were encouraging, confirming not only clinical efficacy, but also the potential to achieve an extended period of disease control, which warrants further trials.
Randomization	This was single-arm, multicenter phase 2 clinical trial involving advanced squamous non-small cell lung cancer (sq-NSCLC) patients who

Randomization	received 2 cycles of nab-paclitaxel/carboplatin and sintilimab, followed by maintenance therapy with sintilimab. No randomization has been performed.
Blinding	Because this was a single arm study, blinding was not necessary.

Reporting for specific materials, systems and methods

We require information from authors about some types of materials, experimental systems and methods used in many studies. Here, indicate whether each material, system or method listed is relevant to your study. If you are not sure if a list item applies to your research, read the appropriate section before selecting a response.

Materials & experimental systems

n/a	Involved in the study	
☐	☒	Antibodies
☒	☐	Eukaryotic cell lines
☒	☐	Palaeontology and archaeology
☒	☐	Animals and other organisms
☐	☒	Clinical data
☒	☐	Dual use research of concern
☒	☐	Plants

Methods

n/a	Involved in the study	
☒	☐	ChIP-seq
☒	☐	Flow cytometry
☒	☐	MRI-based neuroimaging

Antibodies

Antibodies used	Archival or freshly paraffin-embedded tumor tissues were first deparaffinized by a routine histological procedure. The staining followed the instructions supplied with the PD-L1 immunohistochemistry 22C3 pharmDx assay kit (Alilent Technologies, Dako, CA, USA) and were carried out at the central laboratory of the Affiliated Cancer Hospital of Zhengzhou University. Specimens were tested on the platform Dako ASL48.
Validation	The experiments have been performed according to the manufacturer's instructions.

Clinical data

Policy information about clinical studies

All manuscripts should comply with the ICMJE guidelines for publication of clinical research and a completed CONSORT checklist must be included with all submissions.

Clinical trial registration	Chinese Clinical Trial Registry (ChiCTR1900021726).
Study protocol	The study protocol is available as Supplementary Information file.
Data collection	Enrollment took place between May 08, 2020 and April 25, 2022, with a total of 37 patients enrolled at the Affiliated Cancer Hospital of Zhengzhou University / Henan Cancer Hospital. Three patients were enrolled at both the First Affiliated Hospital of Xin Xiang Medical University, and Xin Xiang Central Hospital, two patients were enrolled at both the First People's Hospital of Ping Ding Shan, and the Second People's Hospital of Nan Yang, while one patient was enrolled at the An Yang Cancer Hospital. Data collection and entry/maintenance in eCRF were performed using 91Trial software version 1.0 (EDC system, Shanghai Ashermed Healthcare Communications Co., Ltd, Shanghai, China).
Outcomes	The primary endpoint was PFS, defined as the time from the date of a patient signing informed consent to the date of PD or death. Secondary endpoints included: the ORR; DCR; DOR; OS (time from receipt of signed informed content to death or the last follow-up); and safety. Tumor responses were assessed by the investigator according to the RECIST version 1.1 (Ref 43) every 6 weeks in the first year and every 12 weeks thereafter until PD. Safety outcomes were measured by the prevalence of TRAEs, which were graded based on NCI Common Terminology Criteria for Adverse Events (CTCAE) version 5.0. Changes in vital signs, physical examination results and laboratory tests (such as hematology, clinical biochemistry, urine analysis) before, during and after treatment were carefully recorded and assessed.

Plants

Seed stocks	*Report on the source of all seed stocks or other plant material used. If applicable, state the seed stock centre and catalogue number. If plant specimens were collected from the field, describe the collection location, date and sampling procedures.*
Novel plant genotypes	*Describe the methods by which all novel plant genotypes were produced. This includes those generated by transgenic approaches, gene editing, chemical/radiation-based mutagenesis and hybridization. For transgenic lines, describe the transformation method, the number of independent lines analyzed and the generation upon which experiments were performed. For gene-edited lines, describe the editor used, the endogenous sequence targeted for editing, the targeting guide RNA sequence (if applicable) and how the editor was applied.*
Authentication	*Describe any authentication procedures for each seed stock used or novel genotype generated. Describe any experiments used to assess the effect of a mutation and, where applicable, how potential secondary effects (e.g. second site T-DNA insertions, mosiacism, off-target gene editing) were examined.*

This checklist template is licensed under a Creative Commons Attribution 4.0 International License, which permits use, sharing, adaptation, distribution and reproduction in any medium or format, as long as you give appropriate credit to the original author(s) and the source, provide a link to the Creative Commons license, and indicate if changes were made. The images or other third party material in this article are included in the article's Creative Commons license, unless indicated otherwise in a credit line to the material. If material is not included in the article's Creative Commons license and your intended use is not permitted by statutory regulation or exceeds the permitted use, you will need to obtain permission directly from the copyright holder. To view a copy of this license, visit http://creativecommons.org/licenses/by/4.0/
